# Foam cell-derived 4-hydroxynonenal induces endothelial cell senescence in a TXNIP-dependent manner

**DOI:** 10.1111/jcmm.12561

**Published:** 2015-03-05

**Authors:** Yael Riahi, Nurit Kaiser, Guy Cohen, Ihab Abd-Elrahman, Galia Blum, Oz M Shapira, Tomer Koler, Maya Simionescu, Anca V Sima, Neven Zarkovic, Kamelija Zarkovic, Marica Orioli, Giancarlo Aldini, Erol Cerasi, Gil Leibowitz, Shlomo Sasson

**Affiliations:** a Institute for Drug Research, School of Pharmacy, Faculty of Medicine, The Hebrew UniversityJerusalem, Israel; b Endocrinology and Metabolism Service, Department of Medicine, The Hebrew University-Hadassah Medical CenterJerusalem, Israel; c Department of Cardiothoracic Surgery, The Hebrew University-Hadassah Medical CenterJerusalem, Israel; d Institute of Cellular Biology and Pathology N. Simionescu of The Romanian AcademyBucharest, Romania; e Laboratory for Oxidative Stress, Rudjer Boskovic InstituteZagreb, Croatia; f Department of Pathology, School of Medicine, University of ZagrebZagreb, Croatia; g Department of Pharmaceutical Sciences, Università degli Studi di MilanoMilan, Italy

**Keywords:** atherosclerosis, senescence, foam cells, VEC, 4-HNE, PPARδ, TXNIP

## Abstract

Vascular endothelial cell (VEC) senescence is considered an early event in the development of atherosclerotic lesions. Stressful stimuli, in particular oxidative stress, have been linked to premature senescence in the vasculature. Foam cells are a major source of reactive oxygen species and may play a role in the induction of VEC senescence; hence, we investigated their involvement in the induction of VEC senescence in a co-culture transwell system. Primary bovine aortic endothelial cells, exposed to the secretome of THP-1 monocyte-derived foam cells, were analysed for the induction of senescence. Senescence associated β-galactosidase activity and the expression of p16 and p21 were increased, whereas phosphorylated retinoblastoma protein was reduced. This senescent phenotype was mediated by 4-hydroxnonenal (4-HNE), a lipid peroxidation product secreted from foam cells; scavenging of 4-HNE in the co-culture medium blunted this effect. Furthermore, both foam cells and 4-HNE increased the expression of the pro-oxidant thioredoxin-interacting protein (TXNIP). Molecular manipulation of TXNIP expression confirmed its involvement in foam cell-induced senescence. Previous studies showed that peroxisome proliferator-activated receptor (PPAR)δ was activated by 4-hydroalkenals, such as 4-HNE. Pharmacological interventions supported the involvement of the 4-HNE-PPARδ axis in the induction of TXNIP and VEC senescence. The association of TXNIP with VEC senescence was further supported by immunofluorescent staining of human carotid plaques in which the expression of both TXNIP and p21 was augmented in endothelial cells. Collectively, these findings suggest that foam cell-released 4-HNE activates PPARδ in VEC, leading to increased TXNIP expression and consequently to senescence.

## Introduction

Atherosclerosis is a chronic inflammatory disease affecting certain areas in the arteries, which are characterized by disturbed blood flow 1–3. Impaired function of the vascular endothelium precedes the development of atherosclerotic lesions. Whilst this impairment is part of the natural ageing process 4–6, additional pathophysiological factors promote and accelerate atherosclerosis, among which are hyperglycaemia, dyslipidaemia and hypertension 7–9. These conditions may induce oxidative stress 10, endoplasmic reticulum (ER) stress 11 and inflammation 12,13, which have emerged as major detrimental factors that induce endothelial cell dysfunction.

Cellular senescence has been linked to the development of vascular endothelial cell (VEC) dysfunction in atherosclerosis (reviewed in 14,15). Senescence, which was initially considered part of the ageing process of cultured cells, and termed ‘replicative senescence’, has been linked to progressive shortening of the telomeres due to successive cell divisions (reviewed in 16). Senescence could also result from telomere-independent events, in which mitotically competent cells respond to stress, most often oxidative stress, by growth arrest. In contrast with replicative senescence, stress-induced premature senescence (SIPS) is potentially reversible 17. Senescent cells are characterized by increased expression of the cyclin-dependent kinase inhibitors p21 and p16, and reduced phosphorylated pRb, which lead to cell cycle arrest at the G_0_/G_1_ checkpoint by inactivating members of the transcriptional factor family E2F 18. In addition, senescent cells exhibit increased cell size and increased cytoplasmic activity of senescence-associated β-galactosidase (SA-β-Gal) 14,15. Senescent VEC were observed in areas of atherosclerotic lesions in the human aorta and coronary arteries, and in animal models of atherosclerosis 14,19–23. Cultured VEC were also shown to develop replicative- and stress-induced senescence 24, associated with changes in morphology and gene expression, causing impairment of important cell functions such as endothelium-dependent vasodilation, angiogenesis, vascular healing and increased expression of inflammatory mediators 14,15.

The oxidized form of LDL-cholesterol (OxLDL) plays a central role in the pathophysiology of atherosclerosis, affecting key stages of this process. At the initial stage, it binds to scavenger receptors on VEC surface and induces the expression of adhesion molecules that facilitate monocyte adhesion and migration to the sub-endothelial layer 25. OxLDL transverses the endothelial barrier and enters the sub-endothelium, where it transforms monocytes into macrophages, which become lipid-laden foam cells 26. The latter contribute to the expansion of the lipid core in the atherosclerotic plaque and exacerbate oxidative stress. Indeed, foam cells produce high levels of superoxide, nitric oxide and hydrogen peroxide 27.

Cells exposed to high levels of reactive oxygen species (ROS) often respond by augmenting the generation of 4-hydroxynonenal (4-HNE), the non-enzymatic peroxidation product of n-6 polyunsaturated fatty acids (PUFA) 28–31. In addition, 4-HNE induces the expression of monocyte chemoattractant proteins 32, alters the cell cycle 33,34 and promotes apoptosis 35. Moreover, it may induce ER stress and impair endothelial barrier and coagulation functions 36–38. Cytotoxic effects of 4-HNE are attributed to its covalent interactions with nucleophilic moieties in proteins, DNA and phospholipids 39,40. Excessive accumulation of 4-HNE-modified proteins has been shown to inhibit the proteasome 41. However, recent studies claim that low non-cytotoxic concentrations of 4-hydroxyalkenals, including 4-HNE and 4-hydroxydodecadienal, function as signalling molecules by activating the nuclear receptor peroxisome proliferator-activated receptor (PPAR)δ 31,42–44. Overall, the accumulation of foam cells in the sub-endothelial space may promote atherosclerosis by generating oxidative stress along with accumulation of lipid peroxidation products that affect VEC.

The thioredoxin (TRX) system regulates oxidative stress in various tissues, including the vascular endothelium 45. This antioxidant system consists of TRX, TRX-reductase, NADPH and the natural TRX inhibitor, the thioredoxin-interacting protein (TXNIP) 46. Thioredoxin is ubiquitously expressed in VEC, where it scavenges ROS, prevents apoptosis and promotes angiogenesis 47,48. TXNIP impairs the reducing activity of TRX by interacting with the catalytic site of reduced TRX, leading to increased ROS accumulation 49. Moreover, TXNIP has been shown recently to mediate endothelial cell inflammation in response to disturbed blood flow by increasing monocyte adhesion 50. Lipid peroxidation products, such as 4-HNE and acrolein, inhibit TRX activity by binding to Cys73, which is placed out of the catalytic domain, thus inducing a conformational change; this has been associated with increased ROS production and enhanced monocyte adhesion to VEC 51. These findings suggest that the TRX system is important for VEC function and protects against atherosclerosis.

The present study was designed to investigate the effects of foam cells-derived lipid peroxidation products on VEC senescence. Our findings show that 4-HNE produced by foam cells induces senescence in VEC by activating PPARδ, resulting in increased TXNIP expression.

## Materials and methods

### Materials

All tissue culture media and reagents were purchased from Biological Industries (Beit Haemek, Israel). Transwell plates and inserts were from Nunc (Roskilde, Denmark). Calbiochem (Darmstadt, Germany) supplied GW501516 and 4-HNE; Sigma-Aldrich (Rehovot, Israel) the anti α-tubulin antibody, GSK0660, Oil red O, phorbol 12-myristate 13-acetate (PMA), WY14643 and troglitazone; Abcam (Cambridge, MA, USA) supplied the senescence-associated β-galactosidase detection kit and the following polyclonal antibodies: rabbit anti-p21, rabbit anti-p16 and anti-pRB (phospho-Ser^795^). Mouse anti-TXNIP was from MBL Ltd (Nagoya, Japan) and horseradish peroxidase-conjugated anti-rabbit IgG from Jackson ImmunoResearch (West Grove, PA, USA). Mirus Bio-Corporation (Madison, WI, USA) supplied the TransIT-LT1reagent. Promega (Madison, WI, USA) supplied the dual-luciferase reporter assay system. The TXNIP small-interfering RNA (siRNA) was synthesized by GE Dharmacon (Lafayette, CO, USA). Qiagen (Hilden, Germany) supplied the negative (scrambled) control siRNA (allstars negative control). The pcDNA3 and pEGFP-N1 plasmids were kindly provided by Dr. R. Hertz (The Hebrew University Faculty of Medicine, Jerusalem, Israel). The pSVPORT1-hRXR vector and the 3xPPAR response element (PPRE)-TK-luciferase plasmid were courtesy of Dr. B.M. Spiegelman (Dana Farber Cancer Institute, Boston, MA, USA). The pCMX-hPPARγ1 and pCMX-hPPARγ2 plasmids and the respective empty plasmids were kindly provided by Dr. R. Evans (Howard Hughes Medical Institute, La Jolla, CA, USA). Dr. B. Staels (Institut Pasteur de Lille, Lille, France) kindly provided the pSG5 and pSG5-hPPARα vectors. The PPARδ plasmid was prepared by us, as described 44,52. The luciferase reporter construct encoding the human TXNIP promoter region and the plasmid encoding for human TXNIP were kindly provided by Dr. Anath Shalev (University of Wisconsin-Madison, Madison, WI, USA). All primers were synthesized by Sigma-Aldrich. FL-926-A16 was kindly provided by Flamma S.p.A (Chignolo d’Isola, Bergamo, Italy). Organic solvents were from Frutarom (Haifa, Israel) and Mallinckrodt Baker (Deventer, Holland). All chemicals and reagents for LC-MS/MS were of analytical-grade and purchased from Sigma-Aldrich Chemical Co. (Milan, Italy). High-performance liquid chromatography (HPLC)-grade water was prepared with a Milli-Q water purification system. Optimal cutting temperature (OCT) compound for cryosection was purchased from Bar-Naor Ltd. (Ramat Gan, Israel).

### Preparation of oxidized lipoproteins

OxLDL was prepared at the Lipidomics Department, the Nicolae Simionescu Institute of Cellular Biology and Pathology (Bucharest, Romania). Briefly, native LDL was isolated from plasma of healthy donors by ultracentrifugation (Hematology Center, Bucharest, Romania). OxLDL was prepared *in vitro* by incubating nLDL under sterile conditions with 10 μM copper chloride (24 hrs, at 37°C), in the absence of antioxidant protection. The oxidative reaction was stopped by addition of 1 mg/ml EDTA and after extensive dialysis against PBS, pH 7.4, 4°C, oxLDL was stored at 4°C, under sterile conditions. The copper-OxLDL was characterized as previously described 53.

### Cell culture

#### Bovine aortic endothelial cell cultures

Primary cultures were isolated, cultured and characterized as described previously 54. The cells were used up to passage 9, unless otherwise indicated.

#### THP-1 Monocyte

THP-1 cells, purchased from American Type Culture Collection (Rockville, MD, USA), were grown in suspension in complete RPMI 1640 medium according to the supplier’s protocol. To induce differentiation to macrophages, the THP-1 monocytes (10^6^/ml) were exposed to 0.1 μM of PMA for 24 hrs. Foam cell formation was induced by incubating the macrophages with 100 μg/ml of OxLDL for 72 hrs.

#### Transwell cultures

Vascular endothelial cell were plated and grown to confluency in 6-well culture plates using complete DMEM. THP-1 cells were transformed into macrophages or foam cells on transwell insert membranes, as described above. The membranes were then washed three times with fresh DMEM and inserted into the wells over the apical side of the VEC monolayers. The co-culture was continued for the indicated periods. Cell viability was determined by Trypan blue exclusion assay.

### Senescence-associated β-galactosidase assay

The SA-β-Gal activity was determined in VEC monolayers according to the manufacturer’s instructions (Abcam). Briefly, cells were washed, fixed and stained with the X-Gal reagent at pH 6, followed by DAPI staining for visualization of cell nuclei. The percentage of SA-β-Gal positive cells relative to the total DAPI-positive nuclei was assessed by counting an area of 0.55 mm^2^, per images (under ×10 magnifying lens) obtained from three independent experiments using the Nikon confocal microscope software (NIS-Elements AR 4 30.0).

### Western blot analysis

Vascular endothelial cell lysates were prepared and used for Western blot analysis. Briefly, cells were rinsed three times with PBS and lysed with NP-40 lysis buffer (Tris HCl 50 mM pH 7.5, NP-40 1%, sodium deoxycholate 0.25%, EGTA 1 mM, EDTA 1 mM, NaCl 150 mM, sodium orthovanadate 1 mM, sodium fluoride 1 mM, sodium β-glycerophosphate 10 mM, sodium pyrophosphate 5 mM, PMSF 1 mM, protease inhibitor cocktail 1%; Sigma). Bradford assay was used to determine protein concentrations in the lysates. Twenty μg protein samplers were loaded per lane and separated by PAGE and transferred to nitrocellulose membranes. The membranes were blocked with TBST containing 5% (wt/v) bovine serum albumin. For the analysis of TXNIP PBS containing10% (wt/v) low fat dry milk was used for blocking. Incubations with the various antibodies were according to the suppliers’ protocols at the following dilutions: anti-p16 (1:500), anti-p21 (1:1000), anti-phosphorylated pRB (1:1000), anti-TXNIP (1:500), anti-α-tubulin (1:30,000).

### HPLC analysis of 4-HNE

Polar lipids were extracted from macrophage or foam cell culture media and used to determine 4-HNE content by HPLC, as described before 42. Briefly, medium was collected for analysis from confluent cell cultures maintained with serum-free RPMI 1640 medium for 16 hrs. This was important in order to prevent 4-HNE-protein adduct formation. Following extraction of polar lipids and HPLC, the elution peak of 4-HNE (223 nm) was detected at 4.2 min. The recovery of the standard, added to fresh samples prior to extraction, was 85–90%. Peaks in the HPLC profiles were monitored and quantified by using Clarity-Lite software (DataApex Co., Prague, Czech Republic).

### LC-MS/MS analysis

Analysis of FL-926-A16 adducts with 4-HNE, acrolein, glyoxal and methyglyoxal in culture media of THP-1 macrophages or foam cells was performed as described previously 55. The details of the analysis are given in the Supporting information section.

### hTXNIP overexpression

Vascular endothelial cell plated in 6-well dishes were transfected at 60% confluency with the hTXNIP-lacZ expression plasmid or the empty plasmid 56 using TransIT-LT1 reagent, according to the manufacturer’s instructions (Mirus Bio-Corporation). Briefly, DNA (2.5 μg) was complexed with the transfection reagent for 20 min. in OptiMEM medium, then the mixture was added to cells in complete growth medium for an ON incubation. Cells were harvested after 3 days for analysis of TXNIP or after 7 days for analysis of senescence markers.

### siRNA transfection

Vascular endothelial cell plated at 50% confluency in 6-well dishes were transfected with 25 nM of the siRNA for bovine TXNIP or scrambled RNA sequences, using *Trans*IT-TKO (Mirus Bio-Corporation). The RNA sequence was complexed with the transfection reagent for 20 min in OptiMEM and added to cells in complete growth medium. The medium was changed 24 hrs later, and the cells were co-cultured with foam cells in a transwell system for additional 72 hrs, as described above. The cells were then harvested and processed for Western blot analysis. The target sequence for bovine TXNIP silencing was 5′-CAGAAGUUGUCAUCAGUCA-3′.

### Luciferase assay

Vascular endothelial cell cultures, at 60% confluency, were co-transfected with DNA complexed to *Trans*IT-LT1 (Mirus Bio-Corporation) as described above. Analysis of PPAR and TXNIP activation were performed as previously described 44,52. Briefly, PPAR activation was monitored in cells that were transfected with 165 ng of expression vectors for human (h)PPAR-α, hPPAR-γ1, hPPAR-γ2 or hPPAR-δ, along with of the retinoid X receptor (hRXR, 82.5 ng), green fluorescent protein (82.5 ng) and Renilla luciferase (100 ng) expression vectors, and the 3XPPRE-TK-luciferase reporter (500 ng). For analysis of TXNIP activation, cells were co-transfected with a luciferase-reporter construct encoding the human TXNIP promoter region 1777 bp upstream of the ATG start codon (0.5 μg) with renilla luciferase reporter plasmid (0.1 μg). Luciferase-induced luminescence was determined by using the Mithras LB-940 luminometer (Berthold Technologies, Bad Wildbad, Germany); results were normalized to the Renilla luciferase activity, used as an internal control, according to the kit’s instructions.

### Immunofluorescent staining

Human atherosclerotic plaques removed from the carotid bifurcation of patients following endarterectomy and left internal mammary arteries (LIMA), removed during coronary artery bypass graft, were embedded in OCT compound and frozen sections prepared for Immunofluorescence. Cryosections (7 μm thick) were prepared and immunostained with monoclonal antibodies against Factor VIII (1:500; green) and either p21 (1:500, red) or TXNIP (1:100, red) for 16 hrs at 4°C; cell nuclei were visualized with DAPI staining. Secondary antibodies AlexaFluor488- and AlexaFluor647-labelled anti-rabbit and anti-mouse (1:800) were applied for 2 hrs at room temperature. Visualization was by confocal microscopy. The study was approved by the Hadassah Hebrew University Medical Center Institutional Review Board and each patient signed an informed consent.

### Statistical analysis

Data are given as mean ± SEM. One sample Student’s *t*-test was used to validate statistical differences in experiments expressing data as percent of control. Statistical significance of differences between groups was determined by multiple comparison one-way anova, followed by the Dunnett’s test using the GraphPad Prism statistical software.

## Results

### Foam cells induce senescence in VEC

**Figure 1:**
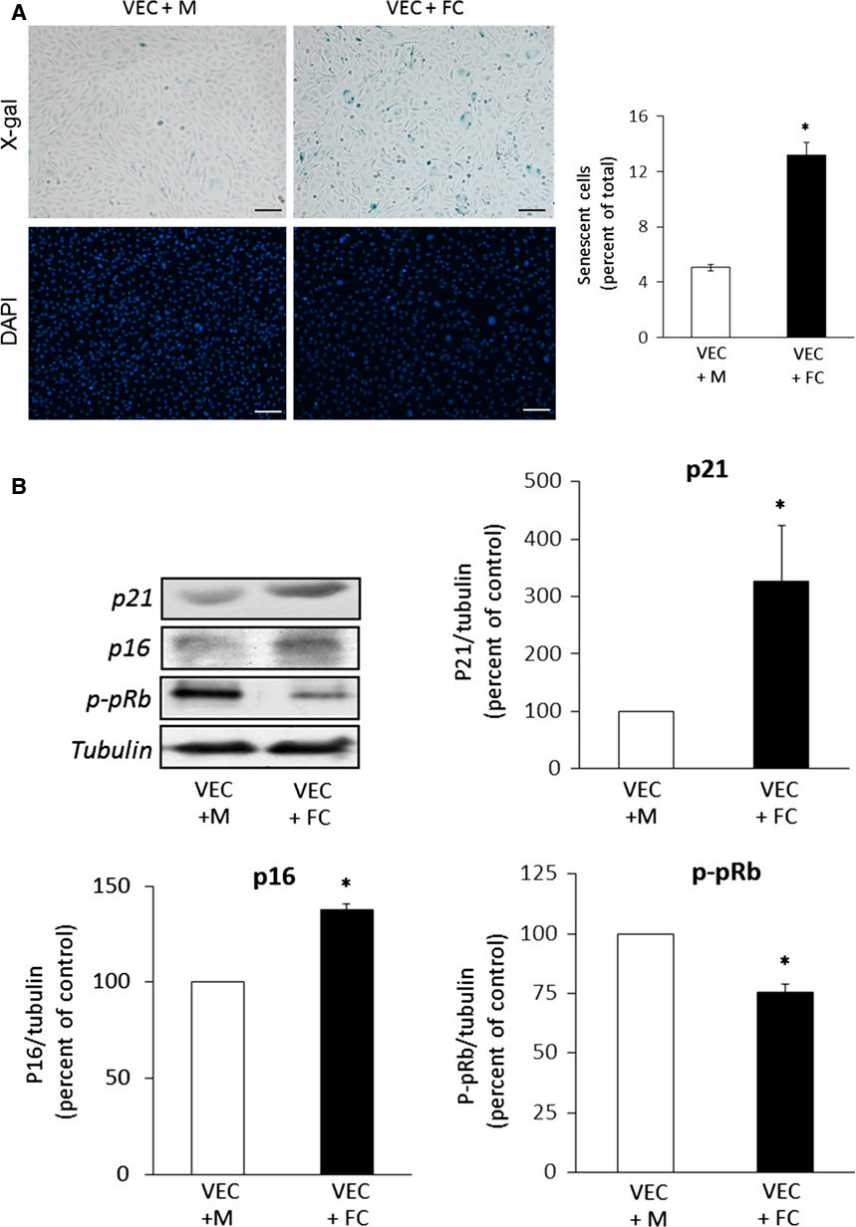
Foam cell-induced senescence in VEC in the transwell co-culture system. (A) Senescence-associated β Galactosidase (SA-β-Gal) activity. VEC and THP-1 macrophages (M) or foam cells (FC) were maintained in transwell co-cultures for 72 hrs, as described under Materials and methods. The VEC monolayers were then washed, fixed and stained with X-Gal or DAPI as described. SA-β-Gal positive cells and total cell number were quantified from three different fields in each well. Results are expressed as percent of SA-β-Gal positive VEC (mean ± SEM, *n* = 3; *P < 0.05 for differences from VEC+M control); scale bar, 100 μm. (B) Expression of senescence markers. At the end of the co-culture period, VEC were harvested, lysed and analysed by Western blot for the expression of p21, p16 and phospho-Ser795-pRB (p-pRB). Shown are representative Western blots and quantification of the different senescence markers. Results are expressed as mean ± SEM, *n* = 3; *P < 0.05, for differences from the respective M-treated VEC (taken as 100%).

The capacity of foam cells to induce senescence in VEC was assessed in an *in vitro* co-culture transwell assay: THP-1 monocytes, seeded on transwell membranes, were first differentiated into macrophages by PMA treatment and then transformed into foam cells following exposure to OxLDL, as described in Materials and methods. Positive Red oil O staining confirmed the formation of lipid-laden foam cells, whereas the control macrophages (PMA-treated only) incorporated negligible amount of the dye (Fig. S1). A significant increase in SA-β-gal staining and the enlarged morphology typical of senescent cells were observed in VEC co-cultured with foam cells (Fig.1A). Western blot analysis (Fig.1B) showed that VEC monolayer co-cultured with foam cells presented increased expression of p21 and p16 and reduced level of phosphorylated (Ser^795^) retinoblastoma protein (p-pRB). Together, these results show the ability of foam cells to induce senescence in VEC *via* secretion of soluble factor(s).

### Senescence is induced by 4-HNE in VEC

**Figure 2:**
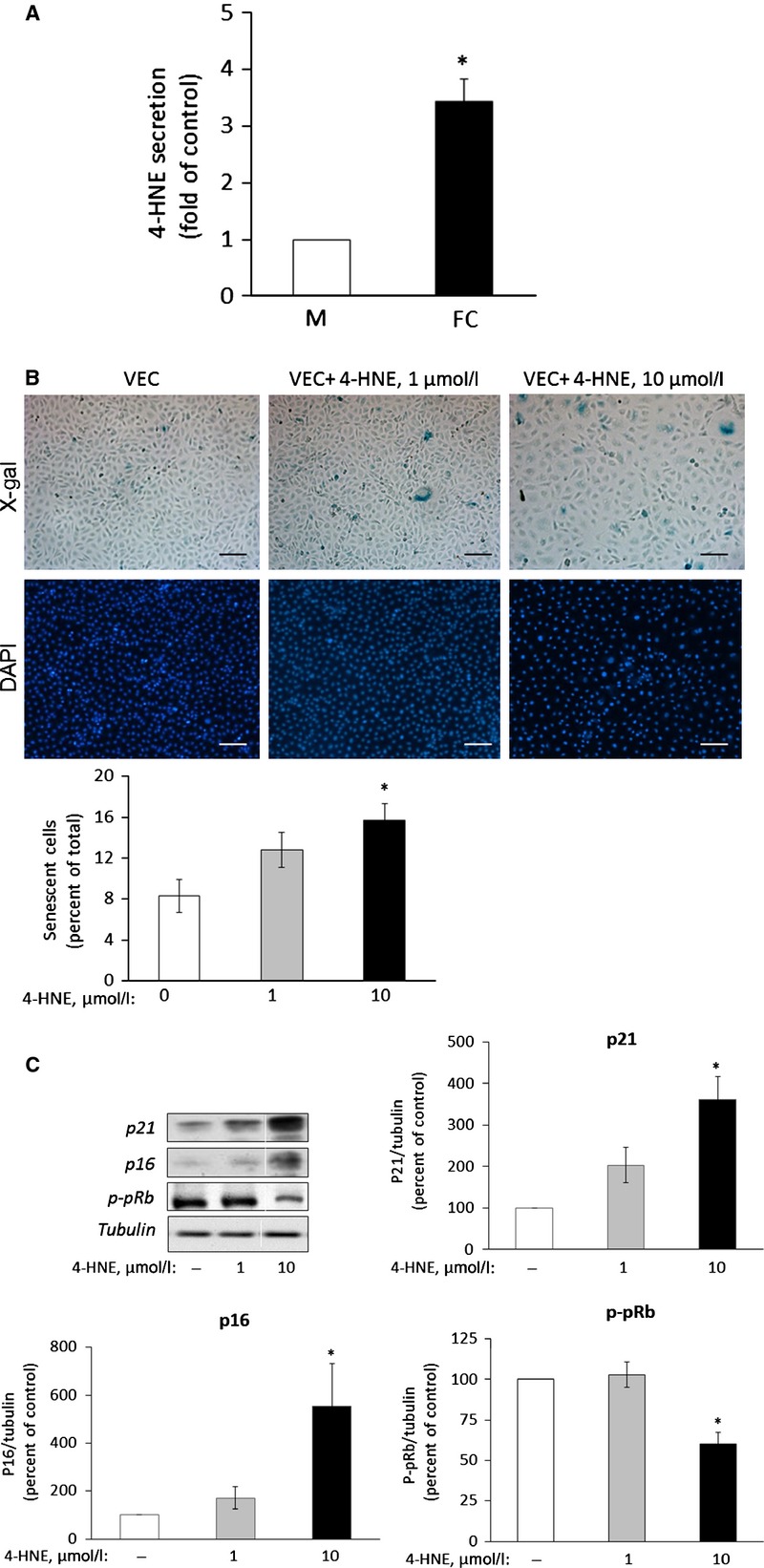
The lipid peroxidation product 4-HNE induces senescence in VEC. (A) THP-1 macrophages (M) and foam cells (FC) were prepared as described under Materials and methods. The culture medium was changed to serum free-medium for 16 hrs. The medium was then collected, cell debris was cleared off by centrifugation and lipids were extracted and taken for HPLC analysis of polar lipids. The level of 4-HNE, normalized to μg of protein, in the M extracts was taken as arbitrary unit (1). Results are expressed as mean ± SEM, *n* = 3. *P < 0.05 for differences from the respective control. (B) Senescence-associated β-Galactosidase (SA-β-Gal) activity in 4-HNE-treated cells. VEC were treated daily with 1 or 10 μM HNE for 7 days. At the end of the incubation period, VEC were processed and analysed as described under the legend of Figure1A. Results are expressed as percent of SA-β-Gal positive VEC (mean ± SEM, *n* = 3; *P < 0.05, for differences from vehicle-treated controls); scale bar, 100 μm. (C) Expression of senescence markers. At the end of the incubation period with 4-HNE, the cells were harvested, lysed and processed as described under the legend of Figure1B. Shown are representative Western blots and quantification of the various markers studied. Results are expressed as mean ± SEM, *n* = 3; *P < 0.05, for differences from the respective 4-HNE untreated controls, taken as 100%.

Next, we studied whether 4-HNE was released from foam cells and could mediate senescence induction. HPLC analysis of polar lipid extracts of media collected from PMA-treated THP-1 cells and from foam-cell cultures showed increased secretion of 4-HNE (3.5 ± 0.4-fold higher than the control, Fig.2A). The effect of exogenously added 4-HNE on senescence in VEC was assessed following exposure to 1 and 10 μM 4-HNE for 7 days. Treatment with 10 μM 4-HNE increased the proportion of senescent cells, demonstrated by cellular enlargement and positive staining for SA-β-gal (Fig.2B). This effect was accompanied by a 40 ± 7.2% reduction in cell number per plate. In addition, increased expression of p16 and p21 and decreased p-pRB were most prominent at this concentration of 4-HNE (Fig.2C). Taken together, these findings suggest that 4-HNE mediates foam cell-induced VEC senescence.

**Figure 3:**
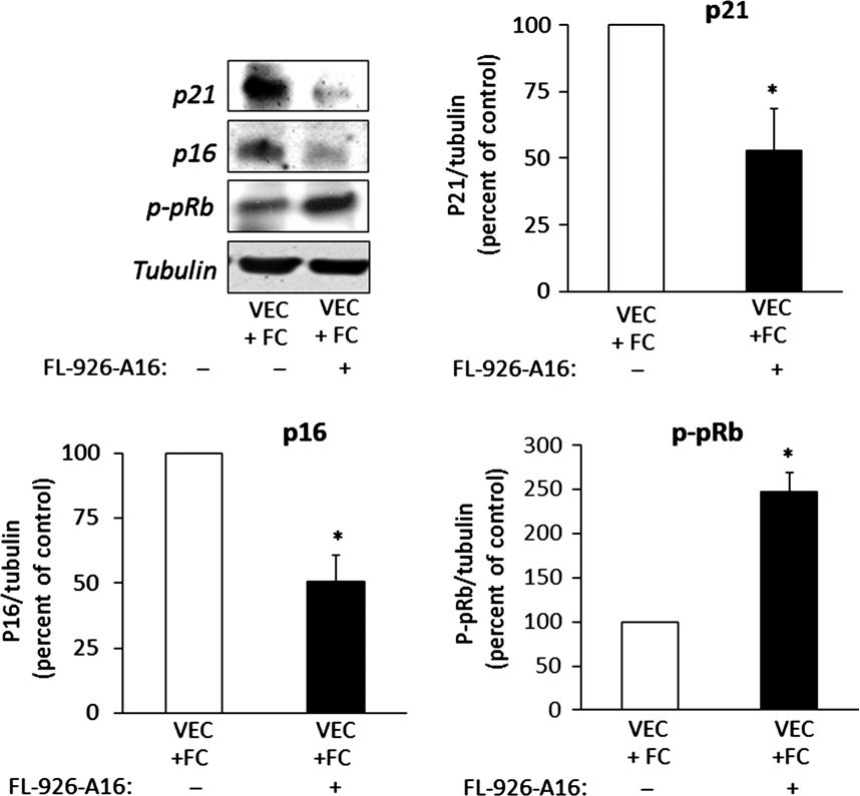
Scavenging of 4-HNE reduces the expression of senescence markers in VEC co-cultured with foam cells. THP-1 monocytes seeded in transwell inserts were transformed to foam cells (FC) as described under legend to Figure1A, and then co-cultured with VEC for 72 hrs, in the absence or presence of 10 mM FL-926-A16, with daily addition of the scavenger. At the end of the incubation period, the cells were harvested, lysed and processed for Western blotting as described under the legend to Figure1B. Shown are representative Western blots and quantification of the various senescence markers. Results are expressed as mean ± SEM, *n* = 3; *P < 0.05 for differences from the untreated cultures.

To further confirm this suggestion, we investigated whether the scavenging of 4-HNE in the transwell system could blunt foam cell-induced VEC senescence. FL-926-A16 is a potent scavenger of bioactive aldehydes, including 4-HNE (European Patent EP 2519507 B1, US Patent 8623900 B2 and 57). Unlike the natural scavenger, L-carnosine, which is degraded by carnosinase in human cells 58, its alcohol derivative FL-926-A16 is resistant to degradation. LC-MS/MS analysis of culture medium from control (PMA-treated) macrophages and foam cells exposed to FL-926-A16 showed that the latter covalently bound 4-HNE (Fig. S2). Interestingly, this analysis also revealed that, in addition to 4-HNE, foam cells secreted glyoxal, methyl glyoxal and acrolein, which were also scavenged by FL-926-A16. When added to the medium of the co-culture system, FL-926-A16 reduced the level of p16 and p21 and increased p-pRB level (Fig.3), supporting the suggestion that the scavenged molecules, including 4-HNE, induce senescence.

### TXNIP mediates 4-HNE-induced senescence

**Figure 4:**
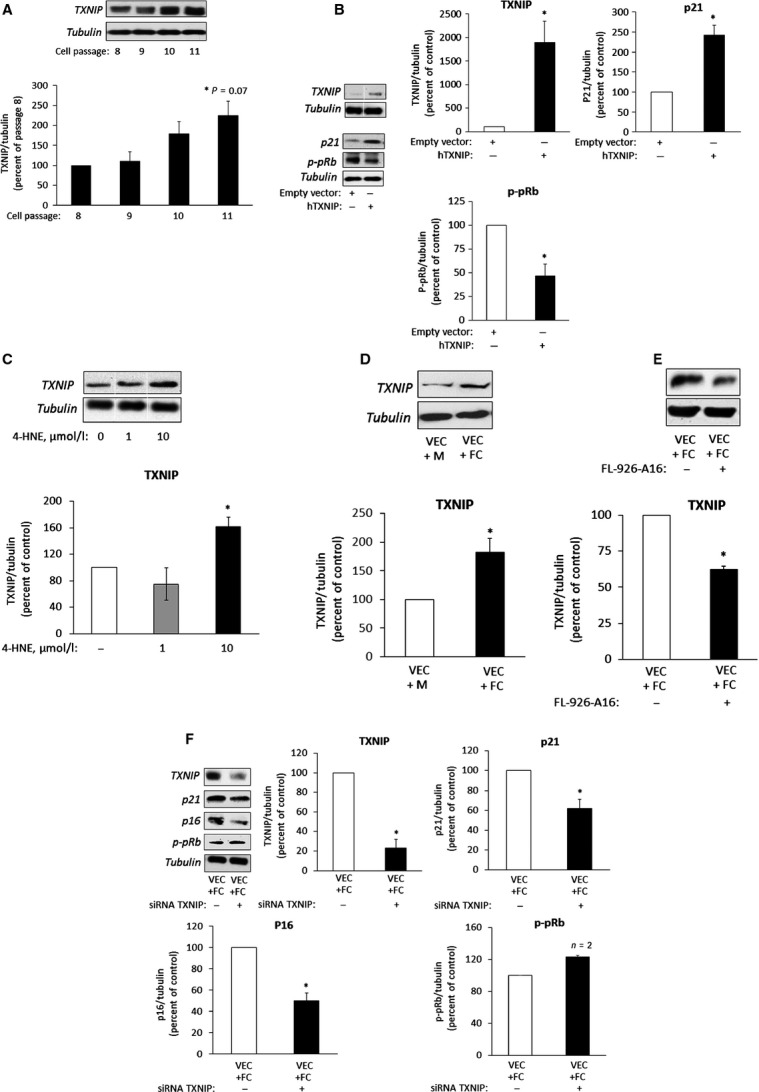
The role of TXNIP in VEC senescence. (A) TXNIP expression in VEC at different passages was analysed by Western blot. *P* < 0.07, for differences from cells at passage 8 (*n* = 3). (B) VEC were transfected with the hTXNIP-lacZ expression plasmid or with an empty vector. Cells were then harvested and taken for Western blot analysis of TXNIP and senescence markers; results are expressed as mean ± SEM, *n* = 3; *P < 0.05 for differences from cells transfected with the empty vector. (C) VEC cultures were treated with the indicated concentrations of 4-HNE for 3 days, with a daily change of medium. Cell lysates were prepared and used for Western blot analysis of TXNIP. The level of TXNIP in untreated controls was taken as 100%. Results are expressed as mean ± SEM, *n* = 3; *P < 0.05 for differences from the control. (D) VEC co-cultured with THP-1 macrophages (M) or foam cells (FC) as described above, and analysed for TXNIP expression. TXNIP expression levels in the VEC + M group was taken as 100%. Results are expressed as mean ± SEM, *n* = 3; *P < 0.05 for differences from the control groups. (E) VEC co-cultured with foam cells (FC) in the absence (control) or presence of 10 mM FL-926-A16, as described under the legend to Figure3, and analysed for TXNIP expression. Results are expressed as mean ± SEM, *n* = 3; *P < 0.05 for differences from the control groups. (F) VEC were transfected with TXNIP siRNA and taken 24 hrs later for co-culture with foam cells (FC) for additional 72 hrs. VEC were then lysed and taken for Western blot analysis of TXNIP or of the senescence markers. The level of TXNIP and the senescence markers in untransfected controls was taken as 100%. Representative Western blots quantification of TXNIP and of various senescence markers are shown. Results are expressed as mean ± SEM, *n* = 3; *P < 0.05 for differences from respective controls (*n* = 3).

It has been suggested that increased TRX activity protects VEC from atherogenesis 59. TXNIP, the natural inhibitor of TRX, plays a prominent role in regulating TRX activity in VEC; high TXNIP levels were associated with endothelial cell dysfunction 49,50,60. Figure4A suggests the involvement of TXNIP in passage-induced replicative senescence in VEC. Indeed, overexpression of TXNIP in low passaged VEC cultures (transfected with the hTXNIP-lacZ expression plasmid) augmented the expression of p21 and reduced p-pRB levels (Fig.4B). Next, we studied the effect of exogenously added 4-HNE on TXNIP expression and its impact on oxidative stress-induced senescence and found increased TXNIP expression in VEC incubated with 10 μM 4-HNE for 7 days (Fig.4C). Importantly, TXNIP expression was increased in VEC co-cultured with foam-cells (Fig.4D). The 4-HNE scavenger FL-926-A16 decreased TXNIP expression in VEC co-cultured with foam cells (Fig.4E), supporting the hypothesis that lipid peroxidation products are involved in the up-regulation of TXNIP expression. Noteworthy, silencing of TXNIP in VEC co-cultured with foam cells prevented the induction of the senescence markers (Fig.4F). Scrambled RNA sequences had no influence on TXNIP and senescence marker expression (Fig. S3). Collectively, these results suggest that 4-HNE up-regulates TXNIP expression and support a role for the latter in both replicative and stress-induced senescence in VEC.

### The role of PPARδ in foam cell-induced senescence

**Figure 5:**
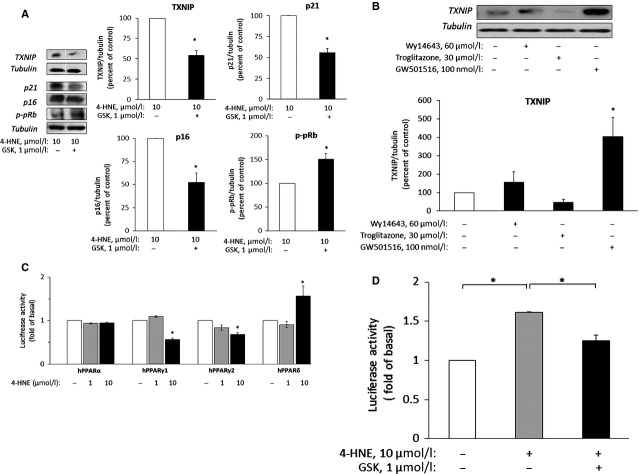
PPARδ modulates the expression of TXNIP and senescence markers in VEC. (A) VEC were treated with 10 μM 4-HNE without (control) or with 1 μM GSK0660 (GSK) for 7 days with daily changes of medium. The expression of TXNIP and the senescence markers was determined in lysed cells by Western blot analysis. The level of TXNIP and the senescence markers in control lysates was taken as 100%. *P < 0.05 for differences from the respective controls (mean ± SEM, *n* = 3). (B) VEC cultures were maintained for 48 hrs without (control) or with the indicated agonists for PPARα, -γ and -δ. Cells were then taken for Western blot analysis of TXNIP expression. *P < 0.05, for differences from control cells treated with vehicle only. (C) VEC cultures were transfected with the following expression vectors: pSG5-hPPARα, pCMX-hPPARγ1, pCMX-hPPARγ2 or pCDNA-hPPARδ and co-transfected with pSVPORT-hRXR, pEGFP-N1 plasmid, 3xPPRE-TK-Luciferase reporter plasmid and Renilla luciferase plasmid. The cells were incubated for 48 hrs. During the last 24 hrs of incubation the cultures were incubated without (control) or with 1 or 10 μM 4-HNE. The cells were then harvested, lysed and the relative luciferase activity was determined and normalized as described under Materials and methods. *P < 0.05, for differences from the respective controls, taken as 1 unit (mean ± SEM, *n* = 3). (D) VEC cultures were co-transfected with a luciferase reporter construct containing the human TXNIP promoter and with the Renilla luciferase reporter plasmid, as described under Materials and methods. After 24 hrs of incubation the medium was changed and cells were incubated for additional 24 hrs without (control) or with 10 μM 4-HNE in the absence or presence of 1 μM GSK0660. Cells were then lysed and taken for the luciferase activity assay as described above. The relative luciferase activity was normalized to untreated control. *P < 0.05, for differences from the indicated groups (mean ± SEM, *n* = 3).

Others and we have shown that 4-HNE activates PPARδ 42,43; hence we investigated whether this mechanism is also involved in 4-HNE-induced senescence in VEC. Figure5A shows that the selective PPARδ antagonist GSK0660 abolished 4-HNE effects on the expression of the different senescence markers and prevented the up-regulation of TXNIP. In addition, VEC were exposed for 48 hrs to selective agonists for the various PPAR receptors at their effective concentrations, as described by us before in the same VEC culture 44. The selective PPARδ agonist, GW501516, markedly augmented TXNIP expression, whereas, the PPARα agonist Wy14643 and the PPARγ agonist, troglitazone, did not modify significantly TXNIP expression (Fig.5B). The hypothesis that 4-HNE activates PPARδ in VEC was further investigated by assessing the transactivation of PPRE-coupled luciferase reporter 44. Briefly, VEC were co-transfected with the PPAR response element (PPRE)-luciferase reporter vector (3xPPRE-TK-Luciferase) and a combination of a specific human PPAR vector and RXR expression vector, followed by a 48 hrs treatment with 4-HNE (Fig.5C). A significant increase in luciferase activity in cells exposed to 10 μM 4-HNE was observed only in cells over-expressing the human PPARδ. The expression of PPARα was not altered in the presence of 4-HNE, whereas the expression of PPARγ_1_ and PPARγ_2_ was somewhat reduced. To further test whether PPARδ mediated the enhancement of TXNIP transcription by 4-HNE, VEC were transfected with the human TXNIP promoter (1777 bp upstream of the ATG start codon)-luciferase vector. TXNIP promoter activity was increased by 4-HNE and reduced in the presence of the PPARδ antagonist GSK0660 (Fig.5D).

### VEC senescence in human carotid atherosclerotic plaques

**Figure 6:**
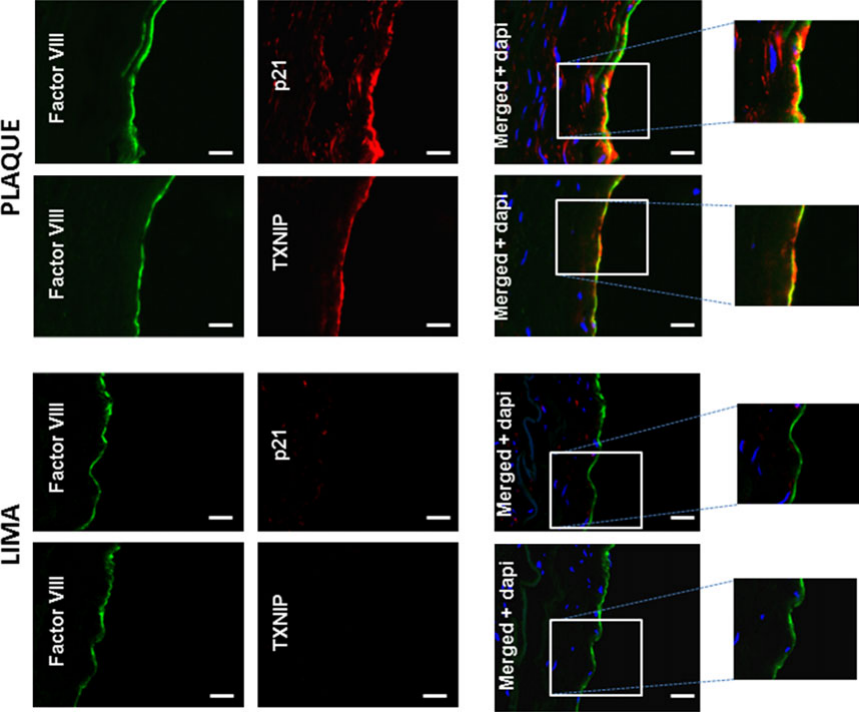
Double immunofluorescence staining for Factor VIII and p21 or TXNIP in sections of human carotid plaque or from LIMA. Human atherosclerotic plaques were removed from the carotid bifurcation of patients by endarterectomy and frozen in OCT as described in Materials and methods. Immunostaining of tissue sections with monoclonal antibodies against Factor VIII (green) and either p21 or TXNIP (red) was performed. Cell nuclei were stained with DAPI. Merged images in the right panels demonstrate co-localization of p21 and TXNIP with Factor VIII (top panels). LIMA sections were used as negative control; scale bar 20 μm. The images are representative of three samples of human carotid plaques and two samples of LIMA.

Finally, we verified the relevance of the above findings to human atherosclerosis. Figure6 shows sections of a human carotid atherosclerotic plaque stained for the endothelial cell marker Factor VIII (green), and p21 or TXNIP (red) in the near-plaque area. The co-localization of TXNIP or p21 with Factor VIII in the shoulder region of the plaque suggests that endothelial cells expressing TXNIP are prone to senescence. No staining of TXNIP or p21 was observed in Factor VIII-positive cells from sections of LIMA, which are resistant to cholesterol buildup and atherosclerosis 61,62 (Fig.6, bottom panels).

## Discussion

**Figure 7:**
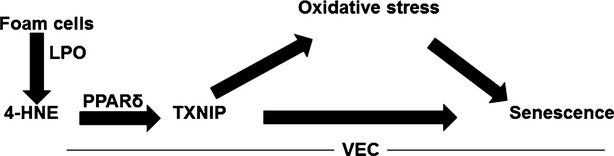
Suggested model of foam cell-induced VEC senescence. Foam cell-derived lipid peroxidation products, among which 4-HNE, reach VEC and activate PPARδ and subsequently increase the expression of TXNIP which augments oxidative stress and VEC senescence.

Cellular senescence has been linked to endothelial cell dysfunction and atherosclerosis 14,63. In the present study we investigated the mechanism involved in foam cell-induced VEC senescence. Using a co-culture system, in which VEC were exposed on the apical side to soluble factors released by foam cells 23,25, we found that the latter produced soluble factors that promoted SIPS in VEC. Our findings provide an important insight into the signal transduction pathway that culminated in endothelial cell senescence: foam cells derived from macrophages following exposure to OxLDL secreted lipid peroxidation products, including the nucleophilic aldehyde 4-HNE. The latter interacted with VEC and activated the nuclear factor PPARδ, which transactivated the TXNIP promoter. Consequently, TXNIP expression was increased, promoting senescence in the endothelial cells (see model in Fig.7).

Non-enzymatic lipid peroxidation of PUFA and their metabolites, promoted by ROS, is known to generate various α,β-unsaturated aldehydes 29,31. Moreover, since these products are often more stable than ROS, they can diffuse into other tissues and propagate oxidative injury. Among these, 4-HNE is considered one of the most abundant and bioactive species. Its plasma concentration in healthy individuals is estimated to be around 1 μM, whereas in disease states it may reach much higher levels 64. Lipid peroxidation products are known to affect the redox balance during ageing and are involved in VEC dysfunctions, including apoptosis, monocyte adhesion to VEC and inflammation; the latter has been suggested recently to result from 4-HNE-induced ER stress 37. Our study shows that exposure of macrophages to OxLDL results in the formation of lipid-laden foam cells that produce several lipid peroxidation products including 4-HNE, which induced VEC senescence at non-cytotoxic concentrations. The stable L-carnosine derivative FL-926-A16, which scavenges lipid peroxidation products, attenuated foam cell-induced VEC senescence. Collectively, these observations suggest that lipid peroxidation products, such as 4-HNE, mediate the effect of foam cells on VEC senescence. Among the known properties of 4-HNE is its biphasic activity: at low concentrations it presents a signalling function *via* activation of the nuclear factor PPARδ, whereas at high concentrations it acts as a cytotoxic agent 31,42,43. Interestingly, our previous studies showed that endothelial cells do not produce detectable amounts of 4-HNE 44. Therefore, it appears that exogenous 4-HNE that diffuses from foam cells is involved in VEC senescence.

An important target of 4-HNE in VEC is the TRX system which is suggested to be involved in the etiology of several human diseases, including atherosclerosis 46. It has been shown before that 4-HNE interacts with and attenuates the function of the TRX system in various cells, including VEC 51,65,66. The present study shows that 4-HNE may also affect the TRX system indirectly by augmenting the expression of its natural inhibitor, TXNIP. Recent studies have shown that TXNIP promoted endothelial inflammation and increased leucocyte adhesion to endothelial cells under conditions of disturbed blood flow that characterize blood vessel regions prone to atherosclerosis 50. The present study shows that increased TXNIP expression in VEC, either following treatment with 4-HNE or by overexpression of human TXNIP, enhanced VEC senescence. Importantly, the silencing of Txnip, which partially inhibited foam cell-induced senescence in VEC, suggests that TXNIP is a downstream target of 4-HNE in this process.

Others and we have previously shown that 4-HNE is an activator of the nuclear factor PPARδ 42,43. Using both pharmacological and molecular tools we now show that PPARδ activation is also involved in 4-HNE-induced VEC senescence. Treatment with 4-HNE of VEC transfected with the different human PPAR isotypes showed specific activation of PPARδ, but not PPARα. Interestingly, 4-HNE reduced the activity of PPARγ1 and γ2. In this context, it should be noted that reduced PPARγ activity has been linked to the induction of senescence in photo-irradiated human fibroblasts 67. In addition, GSK0660, a selective PPARδ antagonist 68, attenuated the effects of 4-HNE on senescence and TXNIP expression. These findings led to the hypothesis that TXNIP is a putative target for PPARδ in VEC. Indeed, the transactivation assay in the presence of 4-HNE and the antagonist GSK0660 support the involvement of PPARδ in this process.

TXNIP has been linked to atherosclerosis by several *in vivo* and *in vitro* studies 50,69. Here, we show by immunofluorescence staining of sections from atherosclerotic arteries that both TXNIP and the p21 senescence marker were present in the endothelial cells near the plaque area, supporting the relationship between TXNIP and senescence *in vivo*. Endothelial cells of LIMA, which are not prone to atherosclerosis, stained neither for TXNIP nor for p21.

What could be the outcome of premature senescence on the vascular endothelium? As opposed to age-related senescence, involving shortening of the telomeres, which is irreversible, telomere-independent SIPS is potentially a reversible process. Brodsky *et al*. 70 have shown that ebselen, a peroxynitrite scavenger and antioxidant, prevented and reversed senescence in early-passaged human umbilical vein endothelial cell cultures. This also occurred in ebselen-fed Zucker diabetic fatty rats, in which the symptoms of vasculopathy were reduced, accompanied by prevention and reversal of endothelial cell senescence in their aorta. Similarly, *N*-acetyl-cysteine reversed stress-induced senescence in bone marrow-derived hematopoietic stem-cells, further supporting the hypothesis that SIPS is reversible 17,71. The association between senescence and atherosclerosis and the potential reversibility of telomere-independent SIPS, as opposed to telomere dependent replicative senescence, suggest that the former may be more relevant in the context of vascular pathology. Therefore, better understanding of the mechanisms of stress-induced senescence is important for devising means to control atherosclerosis by reversing or attenuating this process in VEC. Our study shows that in addition to the well-known effects of oxidative stress, 4-HNE, TXNIP and PPARδ mediate SIPS, and thus may serve as potential targets for the prevention of VEC senescence. The modulation of these targets attenuated foam cell-induced SIPS *in vitro*. Specifically, we showed that attenuation of SIPS could be achieved by: (*i*) scavenging of foam-cell derived lipid peroxidation products, in particular the long-lived bioactive aldehyde 4-HNE; (*ii*) reduction of TXNIP expression and (*iii*) inhibition of PPARδ activity. In conclusion, better characterization of the newly identified pathway(s) of 4-HNE-induced premature senescence in VEC (Fig.7) may provide new insight into the molecular mechanisms of this process and identify potential targets for therapeutic intervention.

## Supporting Information

**Data S1** Foam cell-derived 4-hydroxynonenal induces endothelial cell senescence in a TXNIP-dependent manner.

## References

[b1] Ross R (1999). Atherosclerosis-an inflammatory disease. N Engl J Med.

[b2] Chiu JJ, Usami S, Chien S (2009). Vascular endothelial responses to altered shear stress: pathologic implications for atherosclerosis. Ann Med.

[b3] Chiu JJ, Chien S (2011). Effects of disturbed flow on vascular endothelium: pathophysiological basis and clinical perspectives. Physiol Rev.

[b4] Brandes RP, Fleming I, Busse R (2005). Endothelial aging. Cardiovasc Res.

[b5] Egashira K, Inou T, Hirooka Y (1993). Effects of age on endothelium-dependent vasodilation of resistance coronary artery by acetylcholine in humans. Circulation.

[b6] Hatake K, Kakishita E, Wakabayashi I (1990). Effect of aging on endothelium-dependent vascular relaxation of isolated human basilar artery to thrombin and bradykinin. Stroke.

[b7] Bakker W, Eringa EC, Sipkema P (2009). Endothelial dysfunction and diabetes: roles of hyperglycemia, impaired insulin signaling and obesity. Cell Tissue Res.

[b8] Taddei S, Virdis A, Mattei P (1995). Aging and endothelial function in normotensive subjects and patients with essential hypertension. Circulation.

[b9] Nakamura T, Takano H, Umetani K (2005). Remnant lipoproteinemia is a risk factor for endothelial vasomotor dysfunction and coronary artery disease in metabolic syndrome. Atherosclerosis.

[b10] Singh U, Jialal I (2006). Oxidative stress and atherosclerosis. Pathophysiology.

[b11] Hotamisligil GS (2010). Endoplasmic reticulum stress and atherosclerosis. Nat Med.

[b12] Libby P, Ridker PM, Maseri A (2002). Inflammation and atherosclerosis. Circulation.

[b13] Hansson GK, Robertson AK, Soderberg-Naucler C (2006). Inflammation and atherosclerosis. Annu Rev Pathol.

[b14] Minamino T, Komuro I (2007). Vascular cell senescence: contribution to atherosclerosis. Circ Res.

[b15] Erusalimsky JD (2009). Vascular endothelial senescence: from mechanisms to pathophysiology. J Appl Physiol.

[b16] Hayflick L (2003). Living forever and dying in the attempt. Exp Gerontol.

[b17] Goligorsky MS, Chen J, Patschan S (2009). Stress-induced premature senescence of endothelial cells: a perilous state between recovery and point of no return. Curr Opin Hematol.

[b18] Weinberg RA (1995). The retinoblastoma protein and cell cycle control. Cell.

[b19] Fenton M, Barker S, Kurz DJ (2001). Cellular senescence after single and repeated balloon catheter denudations of rabbit carotid arteries. Arterioscler Thromb Vasc Biol.

[b20] Ota H, Eto M, Kano MR (2008). Cilostazol inhibits oxidative stress-induced premature senescence *via* upregulation of Sirt1 in human endothelial cells. Arterioscler Thromb Vasc Biol.

[b21] Chen J, Goligorsky MS (2006). Premature senescence of endothelial cells: Methusaleh’s dilemma. Am J Physiol Heart Circ Physiol.

[b22] Hayashi T, Yano K, Matsui-Hirai H (2008). Nitric oxide and endothelial cellular senescence. Pharmacol Ther.

[b23] Burrig KF (1991). The endothelium of advanced arteriosclerotic plaques in humans. Arterioscler Thromb Vasc Biol.

[b24] Dierick JF, Eliaers F, Remacle J (2002). Stress-induced premature senescence and replicative senescence are different phenotypes, proteomic evidence. Biochem Pharmacol.

[b25] Bobryshev YV (2006). Monocyte recruitment and foam cell formation in atherosclerosis. Micron.

[b26] Li AC, Glass CK (2002). The macrophage foam cell as a target for therapeutic intervention. Nat Med.

[b27] Rajagopalan S, Meng XP, Ramasamy S (1996). Reactive oxygen species produced by macrophage-derived foam cells regulate the activity of vascular matrix metalloproteinases *in vitro*. Implications for atherosclerotic plaque stability. J Clin Invest.

[b28] Jaganjac M, Tirosh O, Cohen G (2013). Reactive aldehydes–second messengers of free radicals in diabetes mellitus. Free Radic Res.

[b29] Riahi Y, Cohen G, Shamni O (2010). Signaling and cytotoxic functions of 4-hydroxyalkenals. Am J Physiol Endocrinol Metab.

[b30] Cohen G, Riahi Y, Sasson S (2011). Lipid peroxidation of poly-unsaturated fatty acids in normal and obese adipose tissues. Arch Physiol Biochem.

[b31] Cohen G, Riahi Y, Sunda V (2013). Signaling properties of 4-hydroxyalkenals formed by lipid peroxidation in diabetes. Free Radic Biol Med.

[b32] Leonarduzzi G, Chiarpotto E, Biasi F (2005). 4-Hydroxynonenal and cholesterol oxidation products in atherosclerosis. Mol Nutr Food Res.

[b33] Barrera G, Pizzimenti S, Dianzani MU (2004). 4-hydroxynonenal and regulation of cell cycle: effects on the pRb/E2F pathway. Free Radic Biol Med.

[b34] Poot M, Esterbauer H, Rabinovitch PS (1988). Disturbance of cell proliferation by two model compounds of lipid peroxidation contradicts causative role in proliferative senescence. J Cell Physiol.

[b35] Dalleau S, Baradat M, Gueraud F (2013). Cell death and diseases related to oxidative stress: 4-hydroxynonenal (HNE) in the balance. Cell Death Differ.

[b36] Usatyuk PV, Natarajan V (2012). Hydroxyalkenals and oxidized phospholipids modulation of endothelial cytoskeleton, focal adhesion and adherens junction proteins in regulating endothelial barrier function. Microvasc Res.

[b37] Vladykovskaya E, Sithu SD, Haberzettl P (2012). Lipid peroxidation product 4-hydroxy-trans-2-nonenal causes endothelial activation by inducing endoplasmic reticulum stress. J Biol Chem.

[b38] Vatsyayan R, Kothari H, Pendurthi UR (2013). 4-Hydroxy-2-nonenal enhances tissue factor activity in human monocytic cells *via* p38 mitogen-activated protein kinase activation-dependent phosphatidylserine exposure. Arterioscler Thromb Vasc Biol.

[b39] Spickett CM (2013). The lipid peroxidation product 4-hydroxy-2-nonenal: advances in chemistry and analysis. Redox Biol.

[b40] Poli G, Schaur RJ, Siems WG (2008). 4-hydroxynonenal: a membrane lipid oxidation product of medicinal interest. Med Res Rev.

[b41] Petersen DR, Doorn JA (2004). Reactions of 4-hydroxynonenal with proteins and cellular targets. Free Radic Biol Med.

[b42] Cohen G, Riahi Y, Shamni O (2011). Role of lipid peroxidation and PPAR-delta in amplifying glucose-stimulated insulin secretion. Diabetes.

[b43] Coleman JD, Prabhu KS, Thompson JT (2007). The oxidative stress mediator 4-hydroxynonenal is an intracellular agonist of the nuclear receptor peroxisome proliferator-activated receptor-beta/delta (PPARbeta/delta). Free Radic Biol Med.

[b44] Riahi Y, Sin-Malia Y, Cohen G (2010). The natural protective mechanism against hyperglycemia in vascular endothelial cells: roles of the lipid peroxidation product 4-hydroxydodecadienal and peroxisome proliferator-activated receptor delta. Diabetes.

[b45] Altschmied J, Haendeler J (2009). Thioredoxin-1 and endothelial cell aging: role in cardiovascular diseases. Antioxid Redox Signal.

[b46] Mahmood DF, Abderrazak A, El Hadri K (2013). The thioredoxin system as a therapeutic target in human health and disease. Antioxid Redox Signal.

[b47] Yamawaki H, Haendeler J, Berk BC (2003). Thioredoxin: a key regulator of cardiovascular homeostasis. Circ Res.

[b48] Dunn LL, Buckle AM, Cooke JP (2010). The emerging role of the thioredoxin system in angiogenesis. Arterioscler Thromb Vasc Biol.

[b49] Li X, Rong Y, Zhang M (2009). Up-regulation of thioredoxin interacting protein (Txnip) by p38 MAPK and FOXO1 contributes to the impaired thioredoxin activity and increased ROS in glucose-treated endothelial cells. Biochem Biophys Res Commun.

[b50] Wang XQ, Nigro P, World C (2012). Thioredoxin interacting protein promotes endothelial cell inflammation in response to disturbed flow by increasing leukocyte adhesion and repressing Kruppel-like factor 2. Circ Res.

[b51] Go YM, Halvey PJ, Hansen JM (2007). Reactive aldehyde modification of thioredoxin-1 activates early steps of inflammation and cell adhesion. Am J Pathol.

[b52] Shaked M, Ketzinel-Gilad M, Cerasi E (2011). AMP-activated protein kinase (AMPK) mediates nutrient regulation of thioredoxin-interacting protein (TXNIP) in pancreatic beta-cells. PLoS ONE.

[b53] Sima AV, Botez GM, Stancu CS (2010). Effect of irreversibly glycated LDL in human vascular smooth muscle cells: lipid loading, oxidative and inflammatory stress. J Cell Mol Med.

[b54] Kaiser N, Sasson S, Feener EP (1993). Differential regulation of glucose transport and transporters by glucose in vascular endothelial and smooth muscle cells. Diabetes.

[b55] Orioli M, Aldini G, Benfatto MC (2007). HNE Michael adducts to histidine and histidine-containing peptides as biomarkers of lipid-derived carbonyl stress in urines: LC-MS/MS profiling in Zucker obese rats. Anal Chem.

[b56] Minn AH, Hafele C, Shalev A (2005). Thioredoxin-interacting protein is stimulated by glucose through a carbohydrate response element and induces beta-cell apoptosis. Endocrinology.

[b57] Aldini G, Vistoli G, Orioli M (2013). Discovery of FL-926-16, a bioavailable carnosine analogue, effective in metabolic syndrome and diabetes-related disorders. J Diabetes.

[b58] Boldyrev AA, Aldini G, Derave W (2013). Physiology and pathophysiology of carnosine. Physiol Rev.

[b59] El Hadri K, Mahmood DF, Couchie D (2012). Thioredoxin-1 promotes anti-inflammatory macrophages of the M2 phenotype and antagonizes atherosclerosis. Arterioscler Thromb Vasc Biol.

[b60] Yamawaki H, Pan S, Lee RT (2005). Fluid shear stress inhibits vascular inflammation by decreasing thioredoxin-interacting protein in endothelial cells. J Clin Invest.

[b61] Grondin CM, Campeau L, Lesperance J (1984). Comparison of late changes in internal mammary artery and saphenous vein grafts in two consecutive series of patients 10 years after operation. Circulation.

[b62] Loop FD (1996). Internal-thoracic-artery grafts. Biologically better coronary arteries. N Engl J Med.

[b63] Ungvari Z, Kaley G, de Cabo R (2010). Mechanisms of vascular aging: new perspectives. J Gerontol A Biol Sci Med Sci.

[b64] Chapple SJ, Cheng X, Mann GE (2013). Effects of 4-hydroxynonenal on vascular endothelial and smooth muscle cell redox signaling and function in health and disease. Redox Biol.

[b65] Fang J, Holmgren A (2006). Inhibition of thioredoxin and thioredoxin reductase by 4-hydroxy-2-nonenal *in vitro* and *in vivo*. J Am Chem Soc.

[b66] Wakita C, Maeshima T, Yamazaki A (2009). Stereochemical configuration of 4-hydroxy-2-nonenal-cysteine adducts and their stereoselective formation in a redox-regulated protein. J Biol Chem.

[b67] Briganti S, Flori E, Mastrofrancesco A (2013). Azelaic acid reduced senescence-like phenotype in photo-irradiated human dermal fibroblasts: possible implication of PPARgamma. Exp Dermatol.

[b68] Shearer BG, Steger DJ, Way JM (2008). Identification and characterization of a selective peroxisome proliferator-activated receptor beta/delta (NR1C2) antagonist. Mol Endocrinol.

[b69] World CJ, Yamawaki H, Berk BC (2006). Thioredoxin in the cardiovascular system. J Mol Med.

[b70] Brodsky SV, Gealekman O, Chen J (2004). Prevention and reversal of premature endothelial cell senescence and vasculopathy in obesity-induced diabetes by ebselen. Circ Res.

[b71] Zhang X, Li J, Sejas DP (2005). The ATM/p53/p21 pathway influences cell fate decision between apoptosis and senescence in reoxygenated hematopoietic progenitor cells. J Biol Chem.

