# Supplementary material for: Foam cell-derived 4-hydroxynonenal induces endothelial cell senescence in a TXNIP-dependent manner
Source: J Cell Mol Med. 2015 Mar 5;19(8):1887–99. doi: 10.1111/jcmm.12561 (PMC4549039; doi:10.1111/jcmm.12561)
Supplement: Supplementary file 1 [file jcmm0019-1887-sd1.docx]

**ONLINE SUPPORTING INFORMATION**

**Foam cell-derived 4-hydroxynonenal induces endothelial cell senescence in a TXNIP-dependent manner**

**Yael Riahi, Nurit Kaiser, Guy Cohen, Ihab Abd-Elrahman, Galia Blum, Oz M. Shapira, Tomer Koler, Maya Simonescu, Anca V. Sima, Neven Zarkovic, Kamelija Zarkovic, Marica Orioli, Giancarlo Aldini, Erol Cerasi, Gil Leibowitz, Shlomo Sasson^*^**

**SUPPORTING INFORMATION: MATERIALS AND METHODS**

**Oil red O staining**

Oil Red O was used to detect lipid droplets in cells: briefly, cells were fixed with 10% formalin for 1 h then washed with 60% isopropyl alcohol before staining with 0.2% Oil Red O solution. Cells were then examined by light microscopy.

**LC-MS/MS analysis**

The identification of the FL-926-A16 covalent adducts with reactive carbonyl species was carried out by an untargeted HPLC-MS approach, based on the precursor ion scanning (PIS) analysis. PIS analysis was based by setting, as diagnostic product ions, the ions at *m/z* 81.1, 107.1, 178.1, characteristic of FL-926-A16 as well as of the adducts. Structure confirmation of the adducts was achieved by comparing the retention times, molecular weights and MS/MS fragments of the identified adducts with those of the standards prepared by incubating FL-926-A16 with the aldehydes in PBS for 24 h at 37°C. PIS analysis was carried out on a TSQ quantum triple-quadrupole mass spectrometer (ThermoFinnigan Italia, Milan, Italy) with electrospray ionization (ESI) source connected to an HPLC system (Surveyor, ThermoFinnigan Italia, Milan, Italy). Separation was performed on a C-12 Sinergy Polar-RP column (150 mm, 2 mm i.d.; particle size 4 µm) (Chemtek Analytica, Anzola Emilia, Italy) protected by a C12-Polar guard column (4 mm, 2 mm i.d.; 4 µm). The mobile phase (gradient elution) consisted of H_2_O:CH_3_CN: perfluorobutiryc acid 90:10:0.05 (A) and acetonitrile (B) delivered at a flow rate of 0.2 ml/min (injection volume 10 µL). The phase A was maintained at 100% for 5 min, and then the concentration of solvent B linearly increased to 80% B in 17 min, followed by a 5-min isocratic elution (washing period). The composition of the eluent was then restored to the original conditions of 100% A and was re-equilibrated for 5 min. The samples rack and the column compartment were maintained at 4 and 20 ◦C, respectively.

ESI interface parameters were set as follows: middle position; capillary temperature 270ºC; spray voltage 3.5 kV. Nitrogen was used as nebulizing gas. The Q1 quadrupole was scanned from m/z 50 to5 in 1 s (scan time) with resolution of 0.70 m/z, and the precursor ions were fragmented in Q2 using collision potentials equal to 25 V. Finally, Q3 was set to transmit only the ions listed above with a resolution 0.70 m/z. This scan mode was followed by an enhanced resolution experiment for the ions of interest and then by MS/MS acquisitions. MS/MS spectra were acquired using the optimized collision energy (25 V). The HPLC system equipped with a quaternary pump, a Surveyor UV-vis diode array programmable detector 6000 LP, a vacuum degasser, a column compartment and a Surveyor autosampler (200 vials capacity) both with thermostat control, was used for solvent and sample delivery.

**SUPPORTING DATA**

**
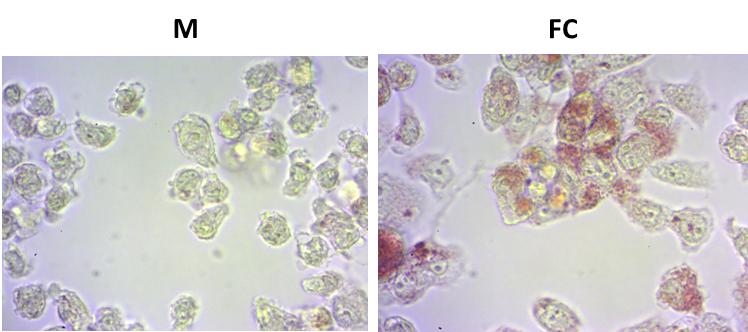
**

**Supplemental Figure 1. OxLDL induces lipid accumulation in foam cells.** THP1 monocytes seeded on transwell membranes (10^6^/well) were differentiated into macrophages (M) by treatment with Phorbol Ester (100 nM) for 24 h in RPMI medium. The cultures were then treated with OxLDL (100µg/ml) for 72 h to induce transformation into lipid-laden macrophages (foam cells, FC). Oil red O staining shows lipid droplets in foam cells.

**
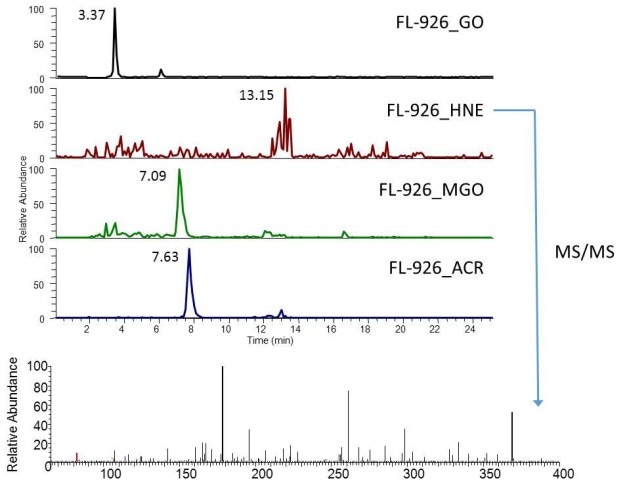
**

**Supplemental Figure 2**.  **Identification of** **FL-926-A16 conjugates in foam cells by LC-ESI/MS**. The upper panels shows the selected ion chromatograms (SIC) reconstituted by setting as filter ions the [M+H]^+^ of the covalent adducts between FL-926-A16 and GO, HNE, MGO and ACR. The [M+H]^+^ values of the covalent adducts were identified in PIS mode. Structure confirmation of the adducts were confirmed by MS/MS analyses (the MS/MS spectrum relative to the covalent adduct between FL-926-A16 and HNE is reported in the lower part of the figure) and by comparing MS/MS spectra of the identified adducts with those of standards prepared by incubating FL-926-A16 with the aldehydes.

**
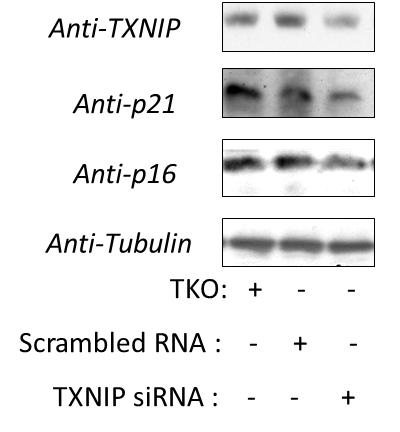
Supplemental Figure 3**.  **Scrambled siRNA and TKO lack silencing effect on TXNIP expression.** VEC were treated with the transfection reagent only (TKO) or were transfected with TXNIP siRNA or with a scrambled sequence and incubated for 72 h. The cells were then lysed and taken for Western blot analysis of TXNIP or the various senescence markers.
